# Large-Scale Differential Gene Expression Transcriptomic Analysis Identifies a Metabolic Signature Shared by All Cancer Cells

**DOI:** 10.3390/biom10050701

**Published:** 2020-04-30

**Authors:** Areej Abu Rmaileh, Balakrishnan Solaimuthu, Mayur Tanna, Anees Khatib, Michal Ben Yosef, Arata Hayashi, Michal Lichtenstein, Yoav D. Shaul

**Affiliations:** Department of Biochemistry and Molecular Biology, The Institute for Medical Research Israel-Canada, The Hebrew University-Hadassah Medical School, Jerusalem 9112001, Israel; areej.abu-rmaileh@mail.huji.ac.il (A.A.R.); solaimut.balakrishna@mail.huji.ac.il (B.S.); mayur.tanna@mail.huji.ac.il (M.T.); aneesk@ekmd.huji.ac.il (A.K.); michal.benyosef1@mail.huji.ac.il (M.B.Y.); arata.hayashi@mail.huji.ac.il (A.H.); michallic@ekmd.huji.ac.il (M.L.)

**Keywords:** cancer metabolism, gene expression analysis, cancer, nucleotide biosynthesis

## Abstract

Cancer-dependent metabolic rewiring is often manifested by selective expression of enzymes essential for the transformed cells’ viability. However, the metabolic variations between normal and transformed cells are not fully characterized, and therefore, a systematic analysis will result in the identification of unknown cellular mechanisms crucial for tumorigenesis. Here, we applied differential gene expression transcriptome analysis to examine the changes in metabolic gene profiles between a wide range of normal tissues and cancer samples. We found that, in contrast to normal tissues which exhibit a tissue-specific expression profile, cancer samples are more homogenous despite their diverse origins. This similarity is due to a “proliferation metabolic signature” (PMS), composed of 158 genes (87 upregulated and 71 downregulated gene sets), where 143 are common to all proliferative cells but 15 are cancer specific. Intriguingly, the PMS gene set is enriched for genes encoding rate-limiting enzymes, and its upregulated set with genes associated with poor patient outcome and essential genes. Among these essential genes is ribulose-5-phosphate-3-epimerase (*RPE*), which encodes a pentose phosphate pathway enzyme and whose role in cancer is still unclear. Collectively, we identified a set of metabolic genes that can serve as novel cancer biomarkers and potential targets for drug development.

## 1. Introduction

Recent advances in cancer research have demonstrated the remarkable complexity of this disease [1]. In addition to the classification based on the tissue of origin, tumors are further characterized into distinct tumor subtypes [2]. These subtypes can then be categorized by the expression of selective markers that, in many instances, have a definite effect on the patient outcomes [3]. However, despite this complexity and heterogeneity, about 20 years ago, a series of shared hallmarks that broadly describes the biology of cancer was proposed [4] and refined a decade later [5]. One such hallmark that is emerging is the recognition of the unique metabolic profile that distinguishes cancer cells from non-proliferating normal tissues [6].

The transition of cells from quiescence to a proliferative state is accompanied by substantial metabolic rewiring. These alterations are thought to enable cells to synthesize sufficient biomolecules to support the highly-demanding replication machinery, as well as to maintain other cellular functions, such as glycan biosynthesis and the oxidative stress response [7]. Cancer-dependent metabolic alteration was characterized almost 100 years ago by Otto Warburg [8] and is still not fully understood. The “Warburg effect” defines the glycolysis pathway as the preferred mechanism for cancer cells to generate ATP [9], rather than the more efficient mitochondrial oxidative phosphorylation pathway [10]. These early findings promoted extensive research over the years, expanding our understanding of cancer-dependent metabolic rewiring. 

Metabolic enzymes bind and modify specific small molecules; thus, their artificial chemical derivatives can potentially serve as competitive inhibitors. Indeed, the earliest set of anticancer drugs are metabolite analogs (antimetabolites) [11], which directly inhibit enzymes essential for cancer cell survival. One of these drugs is methotrexate [12], which targets the folate pathway enzyme, dihydrofolate reductase (DHFR) [13]. Further examples are cytarabine (Ara-C) and 5-fluorouracil (5-FU), which inhibit other nucleotide biosynthesis enzymes [14]. Thus, studying the role of metabolic processes in cancer remains an effective method for developing new classes of anticancer therapeutics, and a means to gain a better understanding of the cellular mechanisms that cause cancer development.

The activity of metabolic enzymes is regulated at multiple levels, including metabolite interaction [15], posttranslational modification by oncogenic signaling pathways [16], and by their transcription level [17]. To systematically analyze the metabolic gene expression profile, we generated a web-based tool (Metabolic gEne RApid Visualizer (MERAV, http://merav.wi.mit.edu)) [18], which provides a means to compare human gene expression between normal tissues, primary tumors, and cancer cell lines. The MERAV web tool was previously applied to identify metabolic genes that function in cancer cell aggressiveness. This analysis resulted in the identification of a “mesenchymal metabolic signature” (MMS), a set of 44 metabolic genes that are upregulated in high-grade tumors with mesenchymal markers [19]. Moreover, a FACS-based shRNA pooled screen demonstrated that several of the MMS gene products are central players in the epithelial–mesenchymal transition (EMT) program. Among them is dihydropyrimidine dehydrogenase (DPYD), the pyrimidine degradation pathway rate-limiting enzyme. DPYD produces dihydropyrimidines (DHPs), which are essential for the proper execution of the EMT program. Identifying the critical role of *DPYD* in the EMT program further reinforced the concept of analyzing metabolic gene expression as a tool to identify uncharacterized cellular mechanisms. However, this analysis was restricted to metabolic gene expression profile in high-grade samples, whereas the identification of signature common to all cancer types is still not fully recognized. 

Here, to understand the global metabolic changes that occur within cancer cells, we analyzed the MERAV database to systematically identify metabolic genes that exhibit a distinct differential expression profile between non-proliferative normal tissues and cancer cells. We found that metabolic gene expression in normal derived samples is heterogeneous, whereby each tissue demonstrates a clear tissue-specific expression profile. However, upon transformation, the samples become more homogenous as they express a common signature designated as the “proliferation metabolic signature” (PMS). This signature includes a set of 87 upregulated and 71 downregulated genes that are enriched in genes encoding for rate-limiting enzymes. Moreover, we identified that the upregulated PMS genes are enriched in essential genes, demonstrating their vital role in cancer cell viability. These findings reveal the presence of a common proliferation signature composed of metabolic genes, which may have future benefits as drug targets and diagnostic markers for cancer.

## 2. Materials and Methods

### 2.1. Median-of-Medians Calculation

In order to calculate the median of normal expression, we first calculated the median of each gene in a given tissue G_tissue_. Following this, we calculated the median of all of the G_tissue_ to get the median-of-medians for each gene (G_all_).

### 2.2. PMS Calculation

The MERAV database contains 16 sample sets, in which the expression patterns of normal tissue, primary tumors, and cancer cell lines originating from the same tissue are presented. The tissues that expressed all three types were identified, and the median of each normal (normal median-of-medians) tissue was determined (Figure S2a). Then, for each tissue, we compared the cancer cell lines’ expression to the normal median-of-medians. The median of all the tissues was combined to one matrix, by which the median value of each gene was then calculated. For each gene, the positive and negative values were separated to generate a score that calculates the median and the number of positive arrays (Figure S2b,c). 

### 2.3. Cell Lines and Cell Culture

The cell lines A549, NCI-H460, NCI-H1395, NCI-H2030, HepG2, SNU-387, and SNU-423 were obtained from ATCC and were maintained in DMEM supplemented with 10% FBS. All cells were cultured at 37 °C with 5% CO_2_.

### 2.4. RNA Preparation and RT-PCR Analysis

Total RNA was isolated from cells using the NucleoSpin^®^ RNA Kit (MACHEREY-NAGEL, Germany), and reverse-transcription was performed using qScript cDNA Synthesis Kit (Quantabio, Beverly, MA, USA). The resulting cDNA was diluted in DNase-free water (1:10) before quantification by real-time quantitative PCR. The mRNA transcription levels were measured using SYBR Green PCR master mix Blue Mix HI-ROX (PCR Biosystems, London, UK) and StepOnePlus (Applied Biosystems, Foster City, CA, USA). All data are expressed as the ratio between the expression level of the target gene mRNA and that for actin. Primers used for qRT-PCR were obtained from Integrated DNA Technology and are listed in Table S8.

### 2.5. Analysis of Different Databases

The Rosario et al. database [20] includes the expression ratio between 24 normal tissues and tumors as provided by the cancer genome atlas (TCGA). For each cancer type, expression profile in the three gene sets (all metabolic genes, PMS upregulated, and PMS downregulated) was determined. Following this analysis, we calculated the mean expression profile of all gene set in each cancer type and presented it as a scatter plot. In addition, we analyzed the “gene expression profiling interactive analysis” (GEPIA, http://gepia.cancer-pku.cn/index.html) [21]. By applying this database, we compared the median expression of the PMS genes between normal and tumors from the same tissue of origin.

### 2.6. Determining the Correlation between the PMS Gene Set and Patient Outcomes

For each member of the PMS gene set (both up and downregulated), we determined the overall survival (OS) using the Kaplan–Meier plotter website (http://kmplot.com/analysis/) [22]. The combined data of all PMS gene set hazard ratio (HR) and their *p*-values are presented as volcano and violin plots. The *p*-value and the HR values were determined by the KM plotter website.

### 2.7. Statistical Analysis and Graphs

All of our statistical analyses, plots, and graphs were generated using R version 3.3.3 or Prism 8 (GraphPad Software, San Diego, CA, USA). The violin plots were generated using the R “ggplot2” library. In each violin plot, a boxplot was added that demonstrates the mean and one standard deviation (SD). 

## 3. Results

### 3.1. Normal Tissues Demonstrate a Tissue-Specific Metabolic Gene Expression Pattern

To gain a comprehensive view of the expression of all the metabolic genes in cancer, we first determined their expression pattern in normal samples [23,24]. The MERAV database contains 726 arrays derived from normal samples, which represent the expression profile of 31 different tissues (Table S1) [18]. This sample number is relatively large and therefore provides a method to analyze the metabolic gene expression in different tissues. The drawback of analyzing normal human tissues is the data collection process, which is mainly performed post-mortem, which can affect the analysis. Thus, we first compared our human gene expression profile to gene expression in mice, as sample collection in mice is faster and more reliable [25,26]. Pearson correlation between mouse and human metabolic gene expression (Figure S1a) demonstrated a statistically significant (*p* < 0.001, Mann–Whitney U test) high correlation (mean = 0.898 ± 0.143) between samples derived from the same tissue relative to the comparison with other tissues (mean = 0.762 ± 0.2) (Figure S1b). This high correlation between the species verified that human metabolic gene expression reflects the actual expression of normal healthy tissues, and reinforces the accuracy of the MERAV database.

**Figure 1:**
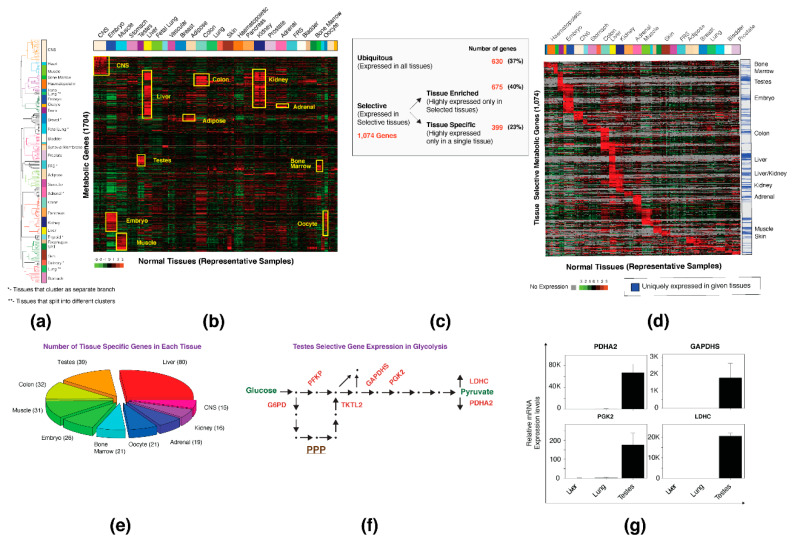
In normal tissues, most metabolic genes demonstrate a selective expression profile. (**a**) Unbiased clustering of metabolic gene expression in normal tissues resulted in tissue-based separation. Each color represents a different tissue. Several of the tissues separated as different dendrogram branches (marked with *). The lung samples separated into two groups (marked with **). CNS—central nervous system, FRS—female reproductive system, UAT—upper aerodigestive tract. (**b**) A specific metabolic gene signature can define normal tissue. A heatmap representing the expression of 1704 metabolic genes in representative normal tissues. All expression levels are relative to the normal median of medians. The top bar represents the normal tissue distribution. (**c**) A table describing the distribution of metabolic genes based on expression profile. (**d**) Metabolic genes demonstrate a tissue-selective expression pattern. Samples with no gene expression are presented as gray. The bar on the right represents the selective and ubiquitous expression pattern. (**e**) A pie chart that represents the expression of tissue specific genes by tissues. All the tissues are determined based on the number of their specific genes. This chart represents the top 10 tissues with the most specific genes; the number of specific genes is indicated. CNS—central nervous system. (**f**) The glycolysis and the pentose phosphate pathway (PPP) contain testis selective isoforms. The testis selective enzymes are in red. Arrows represent an enzymatic reaction; metabolites are presented as dots. (**g**) Gene expression analysis validation. The liver, lung, and testis RNA were converted to cDNA and subjected to quantitative real-time PCR (qPCR). The expression level of all tissues is relative to that of the lung. Each value represents the mean ± SD for *n* = 3.

In order to achieve a clear tissue-selective expression profile, we analyzed the metabolic gene expression pattern in normal human samples. In the MERAV database, the normal samples are represented by 726 arrays that are not equally distributed between the tissues, and therefore can affect the analysis. For example, the central nervous system (CNS) is represented by 149 arrays, whereas the spleen by only five (Table S1). To avoid a skew towards tissues with many samples, we chose a maximum of 10 arrays to represent each tissue expression profile (representative normal arrays) (the exact representative arrays that were used for the analysis are listed in Table S1). These representative arrays were then assembled to a new matrix and subjected to an unsupervised hierarchical clustering analysis (Figure 1a). In addition, we excluded nine tissues that were represented by four or fewer arrays in the analysis (Table S1). We found that most of the samples derived from the same tissues co-cluster together including samples obtained from similar lineages, such as the immune system (bone marrow, hematopoietic, and tonsil), or reproductive system (embryo, oocyte, and testis). However, several of the samples, including the breast tissue, adrenal gland, and thyroid gland clustered separately, suggesting a unique metabolic gene expression pattern (Figure 1a). Interestingly, samples generated from the lung tissue were divided into two groups, one co-cluster with the stomach and the second with the hematopoietic lineage samples (Figure 1a). This co-cluster with hematopoietic lineage conforms with a recent study which reported the presence of hematopoietic progenitor cells in the lung [27]. Importantly, despite the fact that the data was generated from different studies, the samples of the same tissue still clustered together, and this implies the robustness of the MERAV database. 

Next, we defined a selective expression profile of metabolic genes in different tissues. We calculated the median-of-the-medians for each metabolic gene (G_all_) (see Section 2), which resulted in a value representing each gene’s basal expression level. Then, we compared all of the normal representative samples to G_all_ and presented their relative expression as a heatmap (Figure 1b). Based on this heatmap, we subdivided the metabolic genes into three groups (Figure 1c and Table S2): (I) “Ubiquitously expressed genes”, a set of metabolic genes that do not demonstrate any tissue-selective expression pattern (37% of the genes; 630 genes). (II) “Tissue enriched”, metabolic genes that show selective expression in several tissues (40% of the genes; 675 genes) (Figure S1c). (III) “Tissue-specific” metabolic genes that are highly expressed only in a single tissue (23% of the genes; 399 genes) (Figure 1e and Table S3). We referred to tissue enriched and tissue specific groups as the “tissue-selective” gene set, composed of 1074 genes. Furthermore, based on the presence/absence matrix [28], we subdivided this gene set into “uniquely expressed genes” (i.e., genes that are only expressed in a given tissue) and “highly expressed genes” (i.e., genes that are highly expressed in a given tissue) (Figure 1d). Then, we validated our gene expression analysis by correlating our identified tissue-selective genes with known tissue-exclusive metabolic pathways. Therefore, we assigned a corresponding pathway for each gene, as described previously [29], and applied a Fisher’s exact test to determine the enriched metabolic pathways in each tissue (Table S4). For example, the liver selective genes include those encoding known liver-specific metabolic pathways, such as drug metabolism cytochrome P450, amino acid metabolism, fatty acid metabolism, and urea cycle (Table S4). Together, we identified that most of the metabolic genes demonstrate a selective expression pattern, which corresponds to tissue metabolic pathways.

Our analysis identified the testis, after the liver, to be the tissue with the highest number of specific genes (Figure 1e). Interestingly, we found this tissue to selectively express glycolysis/pentose phosphate pathway (PPP) isozymes (Figure 1f) such as pyruvate dehydrogenase alpha 2 (*PDHA2*) [30], glyceraldehyde-3-phosphate dehydrogenase spermatogenic (*GAPDHS*), phosphoglycerate kinase 2 (*PGK2*) [31], and lactate dehydrogenase C (*LDHC*) [32]. We validated this bioinformatic analysis by quantitative polymerase chain reaction (qPCR), which confirmed the selective expression of testis-specific genes (Figure 1g). Together, our analysis recognized the tissue-specific metabolic expression, which can serve as a platform to determine cancer-dependent metabolic rewiring among genes.

### 3.2. Cell Transformation Is Accompanied by a Loss of the Tissue-Specific Expression Profile

**Figure 2:**
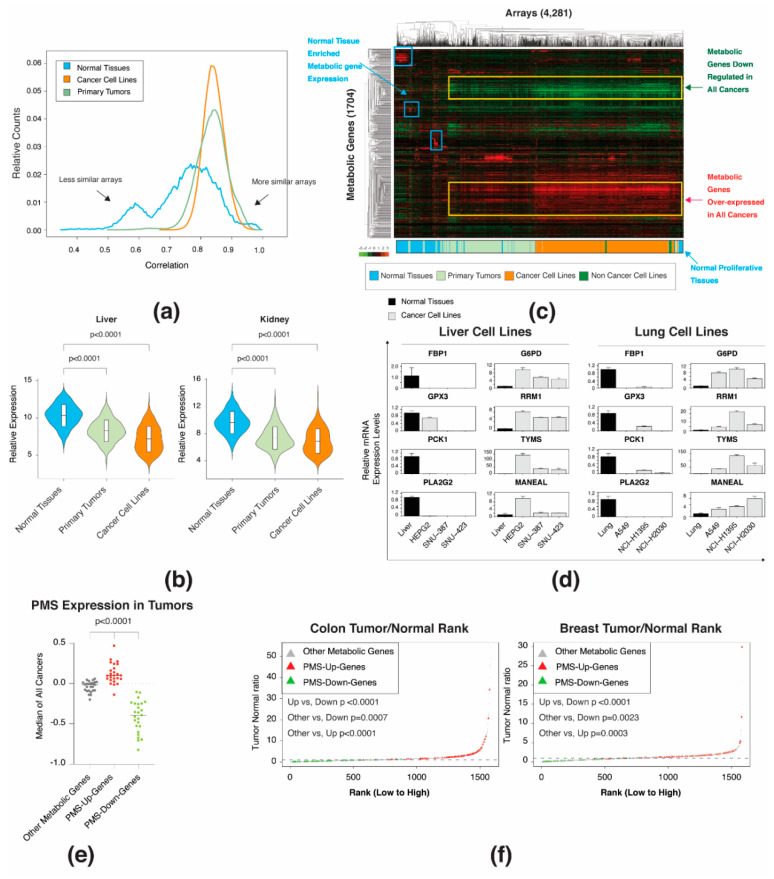
Proliferative cells express a common gene expression signature. (**a**) Metabolic gene expression profile in normal cells is more heterogeneous than in primary tumors and cancer cell lines. In each sample type, the correlation between each array in the metabolic gene expression matrix was calculated. The distribution of the obtained correlation values is presented as a histogram. (**b**) Tissue-specific metabolic gene expression is downregulated in primary tumor and cancer cell lines. The expression profile of liver and kidney tissue-specific genes was compared to primary tumor and cancer cell lines and presented as a violin plot. The boxplot in the violin plot demonstrates the mean value with one SD. The *p*-value was determined by Student’s *t*-test using Prism. (**c**) The selective metabolic gene expression in normal tissues is lost in cancer samples. Two-way hierarchical clustering of the expression levels of 1704 metabolic genes in 4281 different arrays is presented as a heatmap. The bar represents the array distribution based on types. (**d**) Gene expression analysis validation of selected proliferation metabolic signature (PMS) genes in selected tissues and cancer cell lines. RNA was converted to cDNA and subjected to qPCR. The expression level of all tissues and cancer cell lines is relative to the normal liver or lung. Each value represents the mean ± SD for *n* = 3. The *p*-value was determined by Student’s *t*-test using Prism. Left columns in each cancer type are representative of PMS-down, and the right columns are the PMS-up gene set. (**e**) The expression of the PMS gene set was verified utilizing another expression database. The Rosario database includes 24 different tumors, in which the expression profile of tumors samples was compared to the corresponding normal samples. For each tumor type, median ratio of all metabolic genes was calculated. The scatter plot demonstrates the distribution of the median value of each tumor type in the upregulated PMS (PMS-Up-Genes), downregulated PMS (PMS-Down-Genes), and non-PMS (Other Metabolic genes) gene set. The *p*-value was determined by Student’s *t*-test using Prism. (**f**) The PMS up and downregulated gene set was validated using the GEPIA database (http://gepia.cancer-pku.cn/index.html). The expression ratio between tumor and normal tissue was computed for each metabolic gene and ranked from the lowest to the highest. The distribution of upregulated PMS (PMS-Up-Genes), downregulated PMS (PMS-Down-Genes), and non-PMS gene set (Other Metabolic genes) is indicated by different colors. The dashed line indicates that the expression ratio between normal and tumor = 1. The *p*-value was determined by Student’s *t*-test.

The MERAV database contains the expression profile of samples derived from both normal tissues and cancers, which were normalized together [18]. This feature provides a tool to analyze cancer-dependent metabolic gene expression profiles globally. Thus, we first separated the arrays by sample type (normal tissues, primary tumors, and cancer cell lines), and determined the Pearson correlation coefficient of all the samples (Figure 2a). We found that as opposed to normal samples that presented a broad distribution (mean = 0.75 ± 0.1), the distribution of both primary tumors (mean = 0.83 ± 0.05) and cancer cell lines (mean = 0.84 ± 0.03) are narrow relative to normal tissues (standard deviation of 0.1 in normal tissues versus 0.05 and 0.03 in primary tumors and cancer cell lines, respectively). This broad distribution pattern in normal samples supports our finding of tissue-selective genes, as each tissue has a clear and defined expression profile that distinguishes it from other tissues (Figure 1b). However, it also indicates that the transformation from normal tissues to proliferating cancer cells results in samples with similar metabolic gene expression profiles. This loss of tissue identity was further demonstrated in liver and kidney samples, which were chosen since they had the highest tissue-enriched metabolic gene expression (Figure S1c). For both tissue types, we confirmed a significant downregulation in tissue-enriched gene expression in both primary tumors and cancer cell lines (Figure 2b). This finding demonstrates that upon transformation, cells tend to lose their tissue-selective genes and gain a more homogenous expression pattern.

Next, we generated a systematic tool to identify the metabolic gene signature shared by all cancers. Specifically, we compared all the arrays in the MERAV database (normal tissues, primary tumors, and cancer cell lines (4281 arrays)) to the previously calculated basal gene expression values for normal tissues (G_all_) (Figure 1b). Then, we applied hierarchical clustering analysis and identified that the arrays were separated based on the sample type (Figure 2c). As described above (Figure 1b), the normal tissues displayed a clear tissue enriched metabolic expression. In contrast, proliferative samples (primary tumors and cancer cell lines) exhibited a set of metabolic genes that are consistently upregulated or downregulated in all of their arrays (Figure 2c). The location of primary tumor samples between normal tissues and cancer cell lines is attributed to their nature of being a mixture of cancer and normal samples. Interestingly, a small fraction of the normal tissues, including samples mostly derived from embryos, clusters with the cancer cell lines. This suggests that the shared signature is not restricted to cancer but is present in all proliferating cells. 

We isolated the metabolic gene set (Figure 2c) that is upregulated (87 genes) and downregulated (71 genes) throughout all the cancer cell lines and primary tumor arrays (Figure S2a) and designated it as the “proliferation metabolic signature” (PMS). We found that all cancer types, despite their tissue of origin, express the PMS gene set, indicating its essential role in proliferation (Figure S2b–e and Table S5). The proliferation-dependent expression alteration of PMS genes was then individually tested in liver and lung cell lines by qPCR (Figure 2d). We further validated that the PMS gene set is not limited to the MERAV database, as it demonstrated the same expression pattern both in data generated by Rosario et al. [20] (Figure 2e) and by GEPIA (http://gepia.cancer-pku.cn/index.html) [21] (Figure 2f). In addition, by literature search, we identified examples of PMS genes that demonstrated a cancer-dependent alteration in their expression at the protein level (Table 1). Together, our bioinformatics tools identified the PMS dataset, which enables the cancer cells to gain similar expression patterns. Furthermore, the PMS cancer dependent expression pattern was validated by other datasets and at the protein level.

### 3.3. The Proliferation and Cancer-Specific Signature

**Figure 3:**
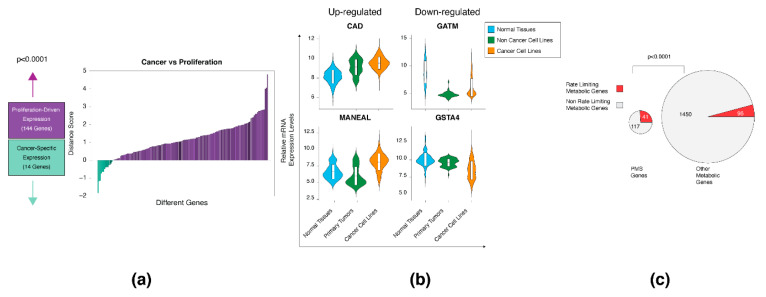
Analysis of the PMS gene set. (**a**) Analysis of the PMS gene set in non-cancer cell lines. Using the mean expression value of each gene in normal (N) tissues, non-cancer (NC), and cancer (C) cells lines, a distance score, defined as the absolute value of (NC-C)-(NC-N), was devised to classify a gene’s expression pattern. Genes exhibiting a proliferation-driven expression signature (distance > 0) were expressed at similar levels in the NC and C groups, whereas cancer-specific genes (distance < 0) were expressed at similar levels in the NC and N groups. The enrichment of genes exhibiting a proliferation-driven signature was quantified using a Fisher’s exact test. (**b**) Violin plots of selected genes with proliferation-driven or cancer-specific expression patterns. Examples of upregulated and downregulated genes in the two gene classes. The boxplot in the violin plot demonstrates the mean value with one SD. (**c**) PMS gene set is enriched in rate-limiting enzymes. A pie chart representing the proportion of rate-limiting enzymes in the PMS signature versus all other metabolic genes is presented. The *p*-value was determined by Student’s *t*-test using Prism.

The normal proliferative samples clustered at the edge of the cancer cells’ array (Figure 2c), suggesting that several of the PMS genes are proliferation-dependent and not unique to tumors. To identify these candidates, we took advantage of our large-scale gene expression database that includes the expression of 79 non-cancer cell lines [18]. These cell lines contain the human breast epithelial cell line MCF10A, the vascular endothelium cell line HUVEC, the testis cell lines HS-1-T, primary human osteoblast, bone marrow, and mesenchymal stromal cells. In order to systematically identify the PMS genes that are cancer-specific, we first determined the expression of each gene in normal tissues (N) and cancer cell lines (C). Then, we defined the expression of the same gene in the non-cancer cell lines (NC) and compared the distribution with that of C as well as N. Accordingly; we determined a distance score, which is defined by the absolute value (NC-C)-(NC-N) for each gene of the universal signature. Genes that demonstrate expression changes in both cancer and non-cancer cell lines (distance > 0) were considered as proliferation-driven expression (Figure 3a and Table S5), whereas genes that demonstrate expression changes only in the cancer cells (distance < 0) were considered as cancer-specific. By applying this score, we found that 144 genes (91% of the genes) are indeed expressed in both cancer and non-cancer cell lines, whereas 14 genes (9%) demonstrated a cancer-specific signature (Figure 3a).

From the PMS gene set, we selected four representative genes for each group. (i) Carbamoyl-phosphate synthetase 2 (*CAD*), the rate limiting enzyme in the pyrimidine biosynthesis pathway [46], was upregulated in both cancer and non-cancer cell lines. (ii) Glycine amidinotransferase (*GATM*), which catalyzes the rate-limiting step in the synthesis of creatine [47], is downregulated in both cancer and non-cancer cell lines (Figure 3b). (iii) More cancer-specific genes, including mannosidase, endo-alpha-like (*MANEAL*) that plays a role in the N-glycan maturation [48], are overexpressed only in cancer cell lines. (iv) Glutathione S-transferase alpha 4 (*GSTA4*), which is one of the 16 human cytosolic GSTs [49], is downregulated only in cancer cell lines (Figure 3b). Together, we conclude that the PMS is mainly composed of proliferative genes, which are not specific to cancer. However, some PMS genes demonstrated a tumor-dependent expression.

### 3.4. The PMS Gene Set Is Enriched in Rate-Limiting Enzymes

The PMS is composed of genes encoding enzymes belonging to diverse metabolic pathways (Table S6). However, in all of these pathways, only a fraction of these members belongs to the PMS gene set. Thus, we speculated that one of the mechanisms by which cancer cells regulate proliferation is through the activity of the rate-limiting enzymes (RLEs), rather than the upregulation of the entire pathway. These RLEs are characterized by their relatively low rate of catalysis, hence, regulating the metabolic flux [50]. Therefore, we assume that tight control of RLE expression levels can influence the entire metabolic pathway. In humans, there are 149 RLEs [50], but not all of them are found suitable for our analysis, as nine enzymes are demarcated as “non-metabolic” based on our definition, eight enzymes were duplicates, and two (Gamma-Glutamyltransferase 1 (GGT1), and UDP Glucuronosyltransferase Family 1 Member A1 (UGT1A1)) did not pass the array quality control of the MERAV database [18]. We found that the PMS set is enriched in genes that encode to RLEs (41 out of 158) (Figure 3c and Table S7), demonstrating that the proliferation machinery is regulated by the upregulation of selective enzymes in the pathway and not by all the members. These results suggest that focusing on gene-based analysis, specifically on RLEs, rather than on the pathway levels, can lead to the identification of metabolic pathways that are essential for cancer cell proliferation.

### 3.5. The Upregulated PMS Gene Set Is Enriched in Essential Genes

**Figure 4:**
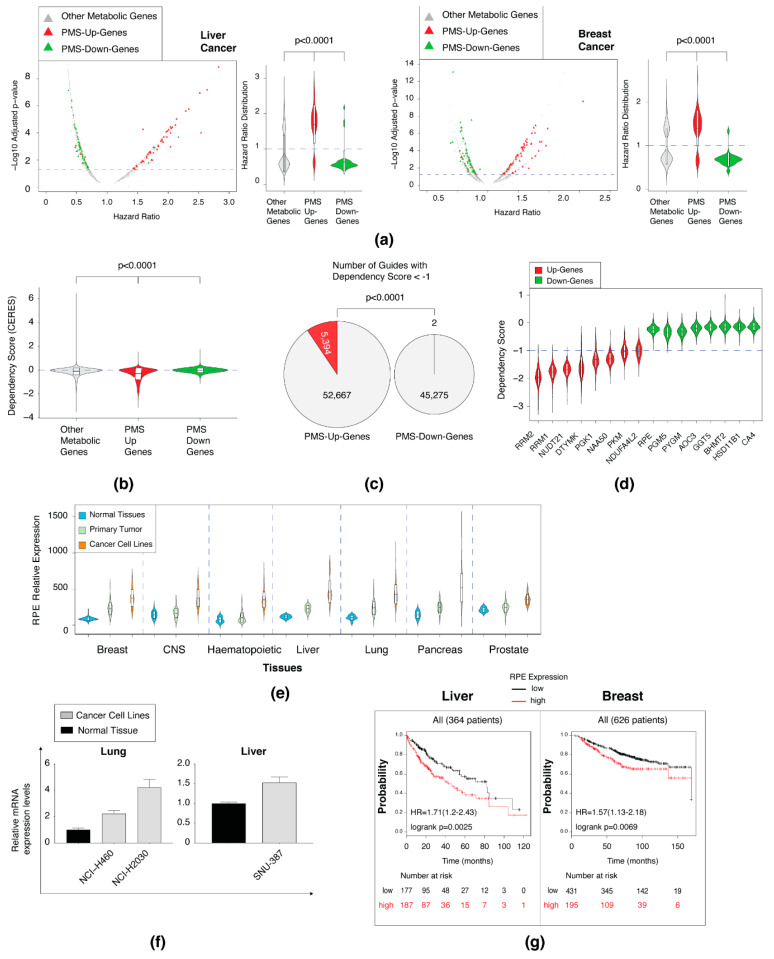
The PMS upregulated gene set is enriched in essential genes. (**a**) The expression profile of the PMS genes corresponds to patient outcomes, as determined by the Kaplan–Meier plotter website (http://kmplot.com/analysis/). The violin plots represent the expression of upregulated PMS (PMS-Up-Genes), downregulated PMS (PMS-Down-Genes), and non-PMS gene set (Other Metabolic genes) that changed significantly. The boxplot in the violin plot of all of the panels demonstrates the mean value with one SD. The *p*-value was determined by Student’s *t*-test using Prism. The dashed line in the volcano plot indicates *p* = 0.05. (**b**) The dependency score distribution of PMS-upregulated genes is lower than the PMS-downregulated genes. The data was collected from the Dependency Map (DepMap) portal (https://depmap.org/portal/). The PMS distribution was compared to non-PMS genes (Other Metabolic genes). The *p*-value was determined by Student’s *t*-test using Prism. The dashed line in the volcano plot indicates dependency score = 0. (**c**) The PMS-Up-Genes are enriched in guides with dependency score less than −1. The DepMap portal defines an essential gene as one whose median dependency score is less than 1. The pie chart represents the number of guides for each group. Red slice is number of guides below −1. (**d**) Comparing the dependency score distribution of selected genes. The PMS upregulated and downregulated genes were ranked based on their average dependency score. The dependency score distribution of top rank of eight upregulated and downregulated genes is presented as violin plots. The dashed line in the volcano plot indicates dependency score = −1 (**e**) RPE expression is elevated in cancer samples relative to normal tissues in multiple cancers derived from different tissues. The distribution of RPE expression in different tissues is presented as violin plots. In each tissue RPE expression was compared between normal tissues (blue), primary tumors (light green), and cancer cell lines (orange). The expression data was obtained from the MERAV webtool. (**f**) Gene expression analysis validation of RPE in selected tissues and cancer cell lines. RNA was converted to cDNA and subjected to qPCR. The expression level of all tissues and cancer cell lines is relative to the normal lung or liver. Each value represents the mean ± SD for *n* = 3. (**g**) RPE expression is associated with poor patient prognosis. Liver and breast patients were divided into two groups (“high” and “low”) according to RPE expression, and their survival rate is presented as Kaplan–Meier survival plots. These plots were generated in the Kaplan–Meier plotter website.

The PMS is composed of metabolic genes that are either upregulated or downregulated in all cancer cell lines. The upregulated gene set is expressed in all cancer cells, suggesting that it can play a central role in tumor survival. Thus, we determined the clinical and biological meaning of the PMS signature by systematically determining their role in patient outcome and on cell viability. We analyzed the Kaplan–Meier Plotter tool (http://kmplot.com/analysis/) [22] for the effect of both the PMS upregulation and the downregulation gene set on patient outcome (for more see Section 2). We found that the PMS-upregulation dataset is significantly enriched with genes and that their overexpression results in poor patient outcome in liver, breast, and lung cancer relative to the PMS-downregulated set (Figure 4a and Figure S3a). Moreover, the mean of the PMS-downregulation dataset KM is below the hazard ratio (HR) of one, indicating the high expression of most of these genes will improve patient outcome, further supporting their role as inhibitors of cell proliferation. Together, we determined a direct correlation between the PMS expression and patient outcome, indicating the role of this gene set in cancer. 

To systemically determine the essential genes in the PMS set, we utilized the DepMap portal (https://depmap.org/portal/) [51,52], as this tool determined gene essentiality in 689 cell lines. First, we differentiated between the upregulated (58,982 guides) and the downregulated (45,277 guides) dependency scores (Figure 4b). This analysis resulted in a significantly lower dependency score of the upregulated gene set (mean = −0.2159) in comparison to the downregulated set (mean = 0.02436). In contrast, knockout of the downregulated gene set resulted in a significant growth advantage (Figure 4b). Notably, the DepMap portal defines that a dependency score of <−1 indicates an essential gene. We combined the dependency scores of all the guides in all cell lines and gene sets and then compared the number of essential hits. This analysis resulted in a distinct difference between the two gene sets as the upregulated set includes 5394 guides that have a <−1 score, and the downregulated set had only 2 (Figure 4c). 

We then tested the dependency score based on genes to identify specific PMS genes that are essential for cell proliferation. We ranked all of the PMS genes and found that the upregulated gene-set contains eight genes that are essential genes (their mean distribution is <−1), which is in comparison to the downregulated genes that were all non-essential (Figure 4d). One of the essential PMS genes is the pentose phosphate pathway (PPP) enzyme ribulose-5-phosphate-3-epimerase (RPE) [53]. The RPE enzyme belongs to the nonoxidative arm of the PPP, and its expression is regulated by KRAS in pancreatic cancers (PDAC) [54]. We found RPE overexpression in many cancer types, including breast, CNS, hematopoietic, liver, lung, pancreatic, and prostate (Figure 4e). We then validated this bioinformatic analysis by (qPCR), which confirmed selective overexpression of RPE in both lung and liver cell lines (Figure 4f). RPE overexpression and its low dependency score predict that its expression affects tumor aggressiveness. Indeed, the high expression of RPE in liver and breast cancer samples correlates with the poor patient outcome as determined by the Kaplan–Meier Plotter tool (http://kmplot.com/analysis/) [22] (Figure 4g). Moreover, in these PDAC cancers, RPE expression is essentially associated with overall survival [55]. Altogether, we conclude that only the upregulated gene set contains essential genes. This result further demonstrates that proliferating cells become addicted to several of the upregulating genes, a characteristic that can be exploited for future identification of anticancer drug targets. 

### 3.6. Mapping the PMS Gene in Selected Metabolic Pathways

**Figure 5:**
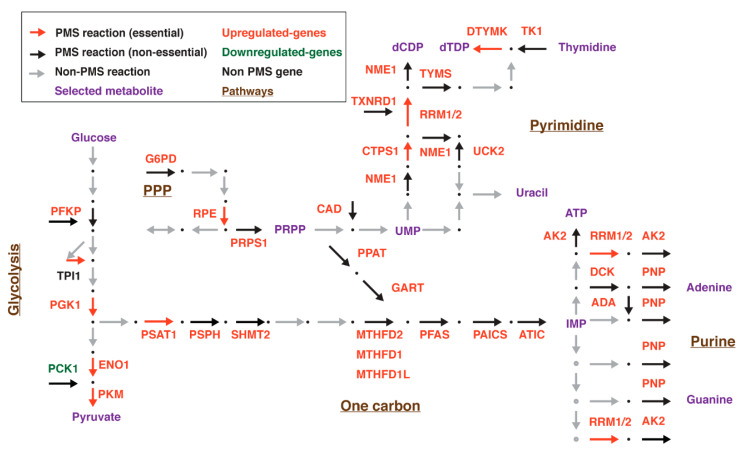
A schematic map demonstrating the PMS genes in selected pathways; (upregulated genes = red; downregulated genes = green). Arrows represent the metabolic reactions; red = essential metabolic PMS reactions; black = non-essential metabolic PMS reactions; gray = non-PMS reactions. Metabolic pathways are named in brown. The essentiality was determined by the DepMap tool (https://depmap.org/portal/).

We generated a map locating the PMS genes in the glycolysis, PPP, one carbon, and nucleotide biosynthesis metabolic pathways. We annotated the genes to indicate the correlation between cancer/proliferation-dependent expression and essentiality (Figure 5). We used the Kyoto Encyclopedia of Genes and Genome (KEGG [56,57]) as well as the HumanCyc (http://humancyc.org, [58]) as references to generate this map. The upregulated gene set includes multiple genes encoding for enzymes belonging to the glycolysis, PPP, one carbon, and nucleotide biosynthesis pathways (Figure 5). However, the downregulated sets comprise genes such as phosphoenolpyruvate carboxylase (*PCK1*). This enzyme belongs to the gluconeogenesis enzyme [59], a pathway which functions opposite to glycolysis. The most abundant metabolic pathway identified in the PMS is nucleotide biosynthesis, composed of 34 genes (Table S6). This gene set is subdivided between the purine and pyrimidine synthesis and to enzymes that catalyze both types of nucleic acid metabolism, such as ribonucleotide reductase isozymes M1 and M2 (RRM1 and RRM2, respectively).

Both purines and pyrimidines can be synthesized by the *de novo* pathways that generate nucleotides from building blocks, which mainly originate from glucose (*de novo* pathway); or through utilizing nucleotides that might be present in the environment (salvage pathway). As indicated in Table S6, members of the *de novo* and salvage pathways generating both purines and pyrimidines are present in the PMS gene set. A more detailed analysis of the nucleotide biosynthesis identified the first enzyme in the *de novo* pathway amidophosphoribosyltransferase (PPAT) as the RLE. This enzyme converts 5-phospho-α-D-ribose 1-diphosphate (PRPP) into 5-phospho-β-D-ribosyl-amine (PRA) through the use of glutamine as an amine donor (Figure 5). This pathway ends with the generation of inosine monophosphate (IMP) that is the precursor for adenosine monophosphate (AMP) and guanosine monophosphate (GMP) synthesis. Interestingly, the majority of enzymes in this pathway are upregulated in cancer, reflecting its importance in the proliferation machinery. 

## 4. Discussion

Major metabolic rewiring accompanies the transformation from resting normal tissues to proliferating cancer cells. Here, we present a systematic analysis of the alteration in metabolic gene expression that is associated with cancer. Specifically, we identified PMS, a set of metabolic genes that are upregulated in all proliferative samples, which is enriched in genes encoding for rate-limiting enzymes. Moreover, we identified that the PMS upregulated gene set has a significant abundance of essential genes. This analysis can serve as a platform for the identification of metabolic genes essential for tumor viability, and therefore can function as a potential tool for the identification of drug targets and cancer biomarkers.

Several studies compared the gene expression profile between normal tissue and cancer cells [17,20,60,61]. These studies mainly emphasize the cancer-dependent changes that are induced in the metabolic pathways, whereas our current study focuses more on the gene levels. Here, we show that the PMS is enriched in RLE, suggesting that modifying the expression of central enzymes in a given metabolic pathway is sufficient to activate it. Thus, if most members in a given metabolic pathway do not demonstrate any cancer-dependent expression changes, but the rate-limiting enzymatic genes do, this can still result in alteration in the metabolic flux. Therefore, while focusing the analysis on metabolic pathways is important, it may wrongly rule out key regulatory elements.

It is well established that the nucleotide biosynthesis pathways are essential for cancer, as many of its members serve as targets of anticancer drugs [11]. Among these targets are the PMS genes *RRM1* and the pyrimidine *de novo* synthesis rate-limiting enzyme thymidylate synthetase (TYMS). Both are well-known drug targets, with multiple molecules aiming to inhibit their function. The existence of a substantial number of genes encoding RLEs and drug targets in the PMS gene set indicates that the analysis can detect established cancer-related metabolic pathways. Therefore, we predict that further studies of the PMS genes would lead to the identification of potential targets for anticancer therapeutics. 

Previously, we identified a set of metabolic genes that are regulated by the EMT program [19] and designated as “mesenchymal metabolic signature” (MMS). There are striking differences between the PMS and the MMS gene sets. As opposed to the PMS gene set that is enriched with rate-limiting step enzymes, the MMS has only two. In addition, the PMS is over-represented with genes encoding the enzymes of the nucleotide biosynthesis pathway (24% of the genes in the signature), whereas the MMS encodes only three of these enzymatic genes (5% of the genes in the signature). However, glycan biosynthesis, which was under-represented in the PMS (10% of the genes in the signature), is highly present in the MMS (36% of the genes in the signature). Thus, the universal signature is composed of genes that are important for the generation of building blocks that serve the proliferation machinery. The mesenchymal signature is mainly composed of genes that function in the generation of the extracellular matrix.

## 5. Conclusions

This current study identifies the differential gene expression pattern of normal tissue vs. cancer samples. Specifically, a set of 158 genes that encode for enzymes in multiple metabolic pathways were identified to be differentially regulated in all proliferative cells. This set designated as proliferation metabolic signature (PMS), is enriched in rate-limiting enzymes, and in essential genes, suggesting it can function as a platform for identification of unknown cell vulnerabilities that can be exploited for the development of novel anticancer drugs.

## Figures and Tables

**Table 1:** Cancer-dependent expression profile of selected PMS encoding proteins. ribonucleotidereductase isozymes M1 and M2 (RRM1 and RRM2, respectively), Phosphoglycerate Kinase (PGK1), Deoxythymidylate Kinase (DTYMK), Pyruvate Kinase Muscle (PKM), Phosphoglucomutase 5 (PGM5), Glycogen Phosphorylase, Muscle Associated (PYGM), Amine Oxidase Copper Containing 3 (AOC3), Betaine-Homocysteine Methyltransferase (BHMT2), Carbonic Anhydrase IV (CA4), Hydroxysteroid 11-Beta Dehydrogenase 1 (HSD11B1).

Enzyme Name	PMS Gene Set	Cancer Type	Reference
RRM1	Upregulated	Lung cancer	[33]
RRM2	Upregulated	Lung, gastric, uterine cervix, glioma	[33,34,35,36]
PGK1	Upregulated	Pancreatic ductal adenocarcinoma (PDAC)	[37]
DTYMK	Upregulated	Non-small cell lung cancer	[38]
PKM	Upregulated	Colorectal cancer	[39]
PGM5	Downregulated	Colorectal cancer	[40]
PYGM	Downregulated	Breast cancer	[41]
AOC3	Downregulated	Colorectal cancer	[42]
BHMT2	Downregulated	Hepatocellular carcinoma	[43]
CA4	Downregulated	Colorectal cancer	[44]
HSD11B1	Downregulated	Hepatocellular carcinoma	[45]

## Supplementary Materials

The following are available online at https://www.mdpi.com/2218-273X/10/5/701/s1, Figure S1: In normal tissues, most metabolic genes demonstrate a selective expression profile, Figure S2: Determining the PMS gene set, Figure S3: The PMS-up set is enriched with essential genes, Table S1: Each normal tissue is represented by a different number of arrays, Table S2: Summarizing the expression pattern of each metabolic genes, Table S3: Distribution of tissue-selective genes in each tissue, Table S4: Elevated metabolic pathways within each normal tissue, Table S5: Distance score calculation, Table S6: Defining the PMS gene set based on their metabolic pathways, Table S7: Defining the PMS genes that encode for rate-limiting enzymes, Table S8: A list of the used qPCR primers.

## References

[1] Hoadley K.A., Yau C., Hinoue T., Hinoue T., Wolf D.M., Lazar A.J., Drill E., Shen R., Taylor A.M., Cherniack A.D. (2018). Comprehensive Characterization of Cancer Driver Genes and Mutations. Cell.

[2] Waks A.G., Waks A.G., Winer E.P., Winer E.P. (2019). Breast Cancer Treatment: A Review. JAMA.

[3] Hanahan D., Weinberg R.A. (2000). The Hallmarks of Cancer. Cell.

[4] Hanahan D., Weinberg R.A. (2011). Hallmarks of Cancer: The Next Generation. Cell.

[5] Zhu J., Thompson C.B. (2019). Metabolic regulation of cell growth and proliferation. Nat. Rev. Mol. Cell Biol..

[6] Cantor J.R., Sabatini D.M. (2012). Cancer cell metabolism: One hallmark, many faces. Cancer Discov..

[7] Warburg O. (1956). On the Origin of Cancer Cells. Science.

[8] Lee N., Kim D. (2016). Cancer Metabolism: Fueling More than Just Growth. Mol. Cells.

[9] Vander Heiden M.G., Cantley L.C., Thompson C.B. (2009). Understanding the Warburg effect: The metabolic requirements of cell proliferation. Science.

[10] Tennant D.A. (2010). Targeting metabolic transformation for cancer therapy. Nat. Rev. Cancer.

[11] Farber S., Diamond L.K. (1948). Temporary remissions in acute leukemia in children produced by folic acid antagonist, 4-aminopteroyl-glutamic acid. N. Engl. J. Med..

[12] Kanarek N., Cantor J.R., Lewis C.A., Chan S.H., Abu-Remaileh M., Freinkman E., Schweitzer L.D. (2018). Histidine catabolism is a major determinant of methotrexate sensitivity. Nature.

[13] Erez A., Deberardinis R.J. (2015). Metabolic dysregulation in monogenic disorders and cancer—Finding method in madness. Nat. Rev. Cancer.

[14] Dayton T.L., Jacks T., Vander Heiden M.G. (2016). PKM2, cancer metabolism, and the road ahead. EMBO Rep..

[15] Vander Heiden M.G., Deberardinis R.J. (2017). Understanding the Intersections between Metabolism and Cancer Biology. Cell.

[16] Nanda C.S., Venkateswaran S.V., Patani N., Yuneva M. (2020). Defining a metabolic landscape of tumours: Genome meets metabolism. Br. J. Cancer.

[17] Shaul Y.D., Yuan B., Thiru P., Nutter-Upham A., McCallum S., Lanzkron C., Bell G.W., Sabatini D.M. (2016). MERAV: A tool for comparing gene expression across human tissues and cell types. Nucleic Acids Res..

[18] Shaul Y.D., Freinkman E., Comb W.C., Cantor J.R., Tam W.L., Thiru P., Kim D., Kanarek N., Pacold M.E., Chen W.W. (2014). Dihydropyrimidine accumulation is required for the epithelial-mesenchymal transition. Cell.

[19] Rosario S.R., Long M.D., Affronti H.C., Rowsam A.M., Eng K.H., Smiraglia D.J. (2018). Pan-cancer analysis of transcriptional metabolic dysregulation using The Cancer Genome Atlas. Nat. Commun..

[20] Tang Z., Li C., Kang B., Gao G., Li C., Zhang Z. (2017). GEPIA: A web server for cancer and normal gene expression profiling and interactive analyses. Nucleic Acids Res..

[21] Lanczky A., Nagy Á., Bottai G., Munkácsy G., Szabó A., Santarpia L., Györffy B. (2016). miRpower: A web-tool to validate survival-associated miRNAs utilizing expression data from 2178 breast cancer patients. Breast Cancer Res. Treat..

[22] Wang L., Srivastava A.K., Schwartz C.E. (2010). Microarray data integration for genome-wide analysis of human tissue-selective gene expression. BMC Genom..

[23] Chang C.-W., Cheng W.-C., Chen C.-R., Shu W.-Y., Tsai M.-L., Huang C.-L., Hsu I.C. (2011). Identification of Human Housekeeping Genes and Tissue-Selective Genes by Microarray Meta-Analysis. PLoS ONE.

[24] Miki R., Kadota K., Bono H., Mizuno Y., Tomaru Y., Carninci P., Itoh M., Shibata K., Kawai J., Konno H. (2001). Delineating developmental and metabolic pathways in vivo by expression profiling using the RIKEN set of 18,816 full-length enriched mouse cDNA arrays. Proc. Natl. Acad. Sci. USA.

[25] Su A.I., Wiltshire T., Batalov S., Lapp H., Ching K.A., Block D., Zhang J., Soden R., Hayakawa M., Kreiman G. (2004). A gene atlas of the mouse and human protein-encoding transcriptomes. Proc. Natl. Acad. Sci. USA.

[26] Borges I., Sena I., Azevedo P., Andreotti J., Almeida V., Paiva A., Santos G., Guerra D., Prazeres P., Mesquita L.L. (2017). Lung as a Niche for Hematopoietic Progenitors. Stem Cell Rev..

[27] McClintick J.N., Edenberg H.J. (2006). Effects of filtering by Present call on analysis of microarray experiments. BMC Bioinform..

[28] Possemato R., Marks K.M., Shaul Y.D., Pacold M.E., Kim D., Birsoy K., Sethumadhavan S., Woo H.-K., Jang H.G., Jha A.K. (2011). Functional genomics reveal that the serine synthesis pathway is essential in breast cancer. Nature.

[29] Pinheiro A., Nunes M.J., Milagre I., Rodrigues E., Silva M.J., de Almeida I.T., Rivera I. (2012). Demethylation of the Coding Region Triggers the Activation of the Human Testis-Specific PDHA2 Gene in Somatic Tissues. PLoS ONE.

[30] Danshina P.V., Geyer C.B., Dai Q., Goulding E.H., Willis W.D., Kitto G.B., McCarrey J.R., Eddy E.M., O’Brien D.A. (2010). Phosphoglycerate kinase 2 (PGK2) is essential for sperm function and male fertility in mice. Biol. Reprod..

[31] Zinkham W.H., Blanco A., Clowry L.J. (1964). An unusual isozyme of lactate dehydrogenase in mature testes: Localization, ontogeny, and kinetic properties. Ann. N. Y. Acad. Sci..

[32] Ding Y., Zhong T., Wang M., Xiang X., Ren G., Jia Z., Lin Q., Liu Q., Dong J., Li L. (2019). Integrative Analysis Reveals Across-Cancer Expression Patterns and Clinical Relevance of Ribonucleotide Reductase in Human Cancers. Front. Oncol..

[33] Sun H., Yang B., Zhang H., Song J., Zhang Y., Xing J., Yang Z., Wei C., Xu T., Yu Z. (2019). RRM2 is a potential prognostic biomarker with functional significance in glioma. Int. J. Biol. Sci..

[34] Morikawa T., Hino R., Uozaki H., Maeda D., Ushiku T., Shinozaki A., Sakatani T., Fukayama M. (2010). Expression of ribonucleotide reductase M2 subunit in gastric cancer and effects of RRM2 inhibition in vitro. Hum. Pathol..

[35] Su Y.-F., Wu T.-F., Ko J.-L., Tsai H.-T., Tee Y.-T., Chien M.-H., Chou C.-H., Lin W.-L., Low H.-Y., Chou M.-Y. (2014). The Expression of Ribonucleotide Reductase M2 in the Carcinogenesis of Uterine Cervix and Its Relationship with Clinicopathological Characteristics and Prognosis of Cancer Patients. PLoS ONE.

[36] Hwang T.L., Liang Y., Chien K.Y., Yu J.S. (2006). Overexpression and elevated serum levels of phosphoglycerate kinase 1 in pancreatic ductal adenocarcinoma. Proteomics.

[37] Wang W., Guo Z.H., Lu X., Liao D.J., Peng G.L., Xu X., Yin W.Q., He J.X. (2016). Elevated expression of DTYMK is associated with poor prognosis in patients with Non-small cell lung cancer. Int. J. Clin. Exp. Med..

[38] Christofk H.R., Vander Heiden M.G., Harris M.H., Ramanathan A., Gerszten R.E., Wei R., Fleming M.D., Schreiber S.L., Cantley L.C. (2008). The M2 splice isoform of pyruvate kinase is important for cancer metabolism and tumour growth. Nature.

[39] Sun Y., Long H., Sun L., Sun X., Pang L., Chen J., Yi Q., Liang T., Shen Y. (2019). PGM5 is a promising biomarker and may predict the prognosis of colorectal cancer patients. Cancer Cell Int..

[40] Dieci M.V., Smutná V., Scott V., Yin G., Xu R., Vielh P., Mathieu M.-C., Vicier C., Laporte M., Drusch F. (2016). Whole exome sequencing of rare aggressive breast cancer histologies. Breast Cancer Res. Treat..

[41] Ward S.T., Weston C.J., Shepherd E.L., Hejmadi R., Ismail T., Adams D.H. (2016). Evaluation of serum and tissue levels of VAP-1 in colorectal cancer. BMC Cancer.

[42] Jin B., Gong Z., Yang N., Huang Z., Zeng S., Chen H., Hu S., Pan G. (2015). Downregulation of betaine homocysteine methyltransferase (BHMT) in hepatocellular carcinoma associates with poor prognosis. Tumor Biol..

[43] Zhang J., Tsoi H., Li X., Wang H., Gao J., Wang K., Go M.Y., Ng S.C., Chan F.K.L., Sung J.J. (2016). Carbonic anhydrase IV inhibits colon cancer development by inhibiting the Wnt signalling pathway through targeting the WTAP–WT1–TBL1 axis. Gut.

[44] Liu X., Tan X.-L., Xia M., Wu C., Song J., Wu J.-J., Laurence A., Xie Q.-G., Zhang M.-Z., Liang H.-F. (2015). Loss of 11βHSD1 enhances glycolysis, facilitates intrahepatic metastasis, and indicates poor prognosis in hepatocellular carcinoma. Oncotarget.

[45] Sigoillot F.D., Sigoillot S.M., Guy H.I. (2004). Breakdown of the regulatory control of pyrimidine biosynthesis in human breast cancer cells. Int. J. Cancer.

[46] Sandell L.L., Guan X.-J., Ingram R., Tilghman S.M. (2003). Gatm, a creatine synthesis enzyme, is imprinted in mouse placenta. Proc. Natl. Acad. Sci. USA.

[47] Thompson A.J., Williams R.J., Hakki Z., Alonzi D.S., Wennekes T., Gloster T.M., Songsrirote K., Thomas-Oates J.E., Wrodnigg T.M., Spreitz J. (2012). Structural and mechanistic insight into N-glycan processing by endo-α-mannosidase. Proc. Natl. Acad. Sci. USA.

[48] Hayes J.D., Flanagan J.U., Jowsey I.R. (2005). Glutathione transferases. Annu. Rev. Pharmacol. Toxicol..

[49] Zhao M., Chen X., Gao G., Tao L., Wei L. (2009). RLEdb: A database of rate-limiting enzymes and their regulation in human, rat, mouse, yeast and E. coli. Cell Res..

[50] Dempster J.M., Rossen J., Kazachkova M., Pan J., Kugener G., Root D.E., Tsherniak A. (2019). Extracting Biological Insights from the Project Achilles Genome-Scale CRISPR Screens in Cancer Cell Lines. bioRxiv.

[51] Meyers R.M., Bryan J.G., McFarland J.M., Weir B.A., Sizemore A.E., Xu H., Dharia N.V., Montgomery P.G., Cowley G.S., Pantel S. (2017). Computational correction of copy number effect improves specificity of CRISPR–Cas9 essentiality screens in cancer cells. Nat. Genet..

[52] Patra K.C., Hay N. (2014). The pentose phosphate pathway and cancer. Trends Biochem. Sci..

[53] Ying H., Kimmelman A.C., Lyssiotis C.A., Hua S., Chu G.C., Fletcher-Sananikone E., Locasale J.W., Son J., Zhang H., Coloff J.L. (2012). Oncogenic Kras Maintains Pancreatic Tumors through Regulation of Anabolic Glucose Metabolism. Cell.

[54] Tian G., Li G., Liu P., Wang Z., Li N. (2020). Glycolysis-Based Genes Associated with the Clinical Outcome of Pancreatic Ductal Adenocarcinoma Identified by The Cancer Genome Atlas Data Analysis. DNA Cell Biol..

[55] Kanehisa M., Goto S., Kawashima S., Nakaya A. (2002). The KEGG databases at GenomeNet. Nucleic Acids Res..

[56] Kanehisa M., Goto S., Sato Y., Furumichi M., Tanabe M. (2012). KEGG for integration and interpretation of large-scale molecular data sets. Nucleic Acids Res..

[57] Romero P., Wagg J., Green M.L., Kaiser D., Krummenacker M., Karp P.D. (2005). Computational prediction of human metabolic pathways from the complete human genome. Genome Biol..

[58] Kruiswijk F., Labuschagne C.F., Vousden K.H. (2015). p53 in survival, death and metabolic health: A lifeguard with a licence to kill. Nat. Rev. Mol. Cell Biol..

[59] Peng X., Chen Z., Farshidfar F., Xu X., Lorenzi P.L., Wang Y., Cheng F., Tan L., Mojumdar K., Du D. (2018). Molecular Characterization and Clinical Relevance of Metabolic Expression Subtypes in Human Cancers. Cell Rep..

[60] Satoh K., Yachida S., Sugimoto M., Oshima M., Nakagawa T., Akamoto S., Tabata S., Saitoh K., Kato K., Sato S. (2017). Global metabolic reprogramming of colorectal cancer occurs at adenoma stage and is induced by MYC. Proc. Natl. Acad. Sci. USA.

[61] Gaude E., Gaude E., Frezza C. (2016). Tissue-specific and convergent metabolic transformation of cancer correlates with metastatic potential and patient survival. Nat. Commun..

