# Supplementary figures and images for: Large-Scale Differential Gene Expression Transcriptomic Analysis Identifies a Metabolic Signature Shared by All Cancer Cells
Source: Biomolecules. 2020 Apr 30;10(5):701. doi: 10.3390/biom10050701 (PMC7277211; doi:10.3390/biom10050701)

Normal Tissues

Metabolic Genes

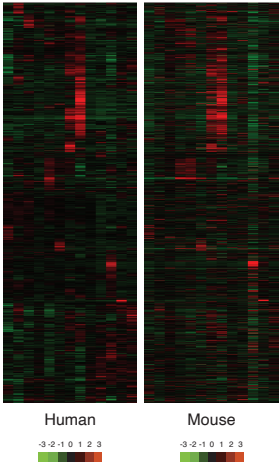

(a)

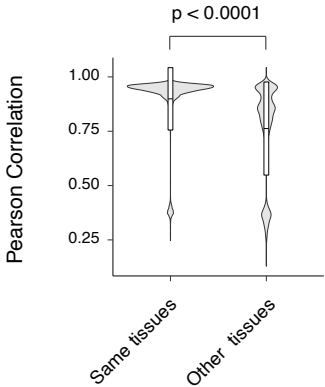

(b)

Number of Enriched genes for Each Tissues

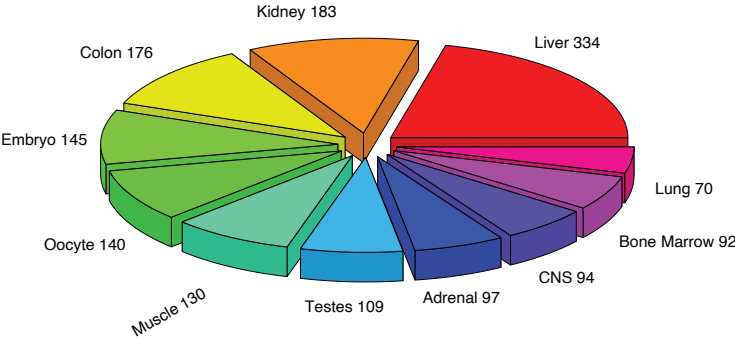

(c)

Supplement: Supplementary file 1 [file biomolecules-10-00701-s001.zip › Figure S1.pdf]

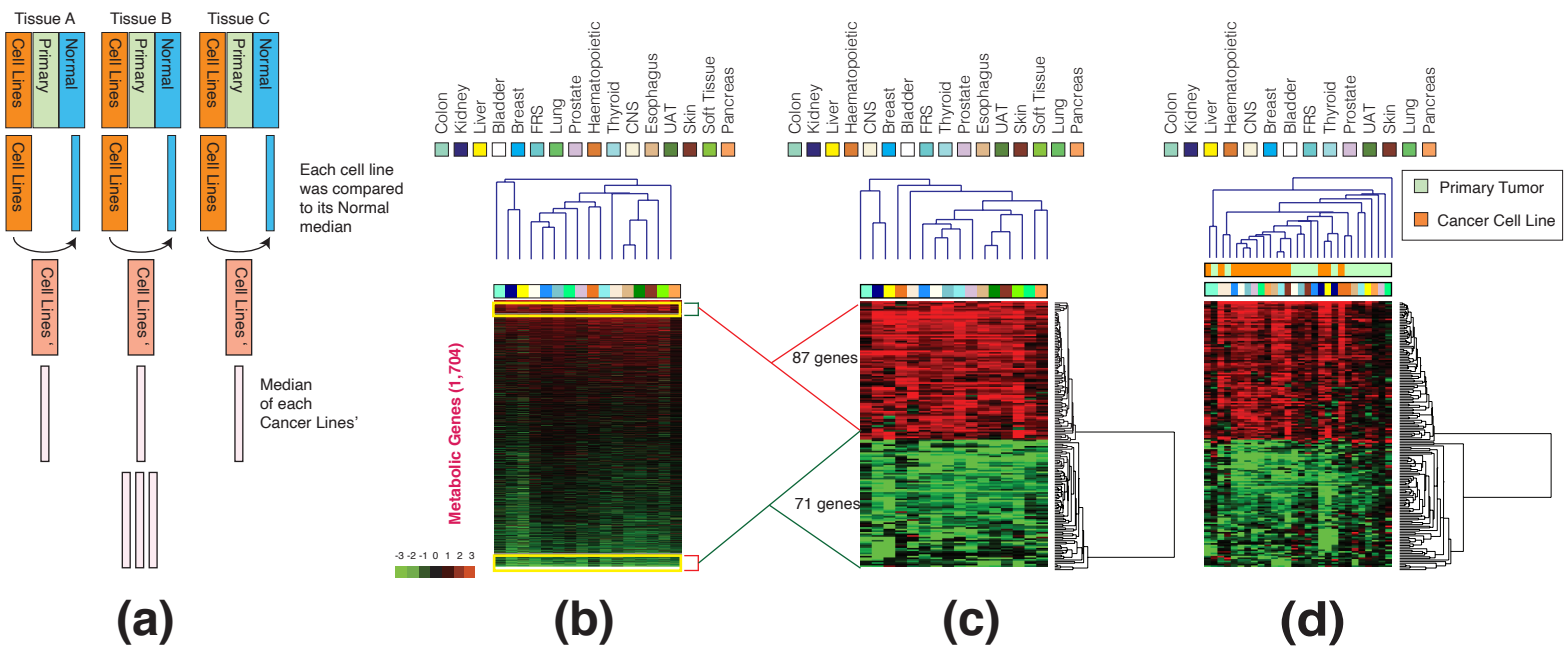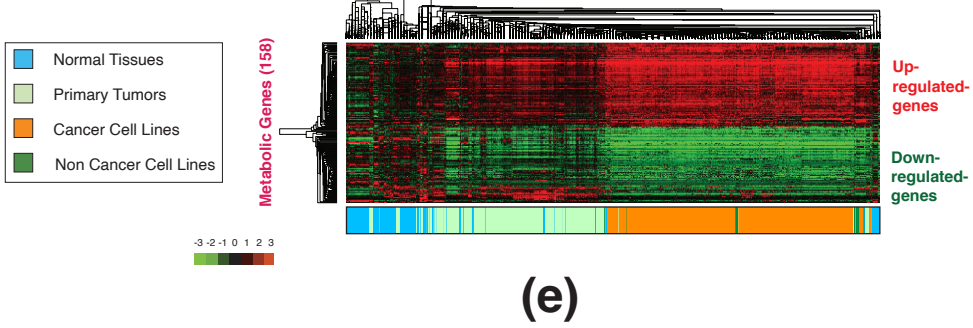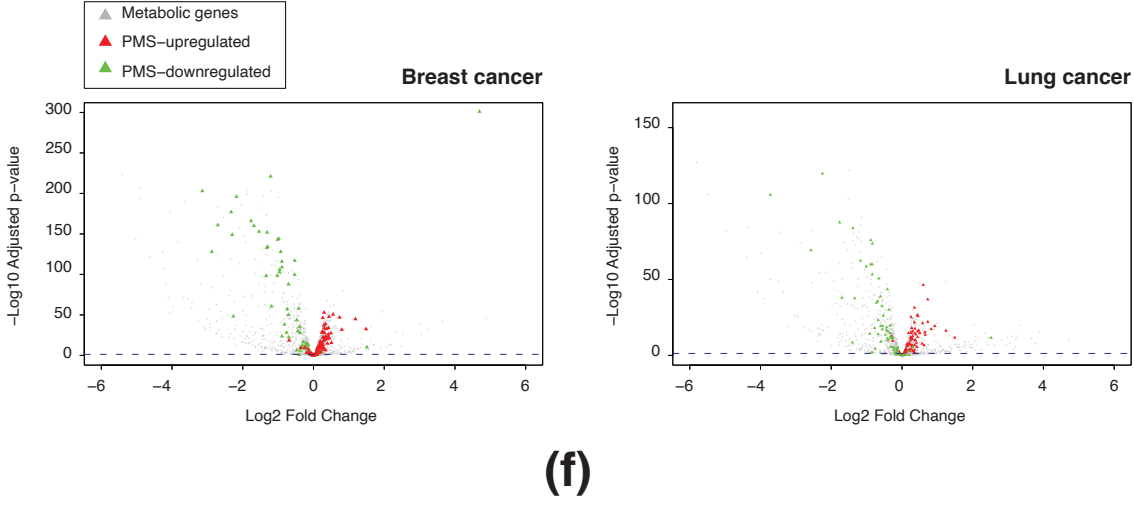

Supplement: Supplementary file 1 [file biomolecules-10-00701-s001.zip › Figure S2.pdf]

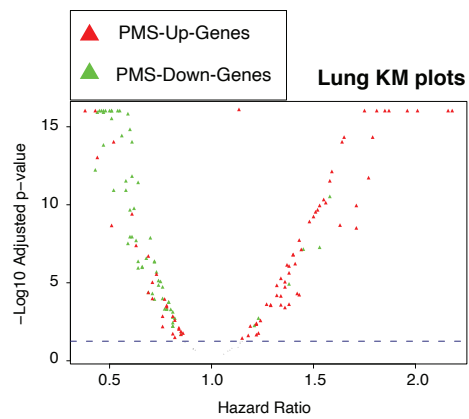

(a)

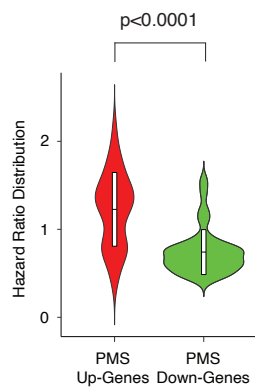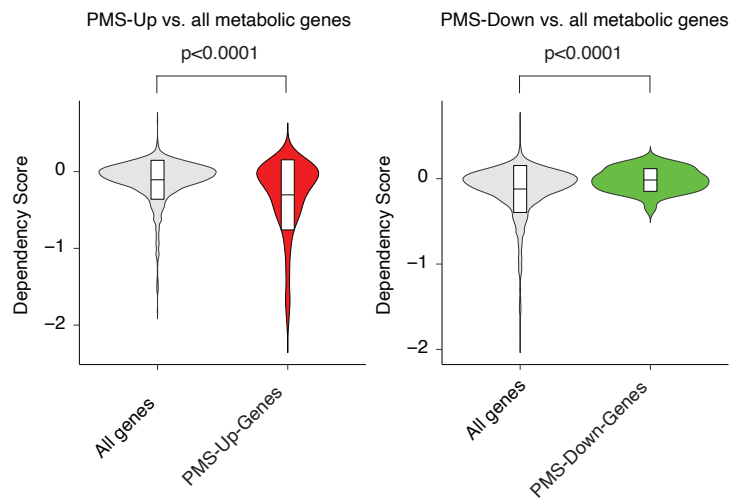

(b)

Supplement: Supplementary file 1 [file biomolecules-10-00701-s001.zip › Figure S3.pdf]
